# Consideration of Two Cases of Ascending Aortic Dissection That Began with Stroke-Like Symptoms

**DOI:** 10.1155/2015/829756

**Published:** 2015-01-18

**Authors:** Chiaki Takahashi, Takashi Sasaki

**Affiliations:** ^1^Department of Neurosurgery, Itoigawa General Hospital, 457-1 Takegahana, Itoigawa, Niigata 941-0006, Japan; ^2^Department of Neurosurgery, Takaoka City Hospital, 4-1 Takara-machi, Takaoka, Toyama 933-8550, Japan

## Abstract

We recently experienced two patients with stroke-like symptoms and ascending aortic dissection (AAD) in our outpatient department. Both patients were transferred to our hospital presenting with neurological deficit such as hemiparesis and conjugate deviation. They did not complain from any chest or abdominal pain. Their MRI did not show fresh infarction or main branch occlusion. A chest CT image showed AAD. The former patient was immediately transferred to a tertiary hospital and the latter received conservative management in the cardiovascular department.* Discussion*. As neither patient was experiencing any pain, we initially diagnosed them with ischemic stroke and began treatment. Fortunately, bleeding complications did not occur. In such cases, problems are caused when intravenous tissue plasminogen activator (t-PA) injection is administered with the aim of reopening the occluded intracranial arteries. In fact, patients with AAD undergoing t-PA injection have been reported to die from bleeding complications without any recognition of the dissection. These findings suggest that confirmation using carotid ultrasound, carotid MR angiography, and a D-dimer test is crucial and should be adopted in emergency departments.

## 1. Introduction

In our emergency department (ED) and outpatient department, when we encounter patients with hyperacute stroke-like symptoms such as hemiparesis, aphasia, and conjugate deviation of the eyes, we first assess whether intravenous tissue plasminogen activator (t-PA) injection can be used as a treatment. We then perform a head computed tomography (CT) scan, blood test, chest X-ray, evaluation of NIH Stroke Scale (NIHSS), and electrocardiogram (ECG) test. Finally, we determine whether intravenous t-PA injection is indicated based on these results and check the items defined by The 2009 Japanese Guideline for the Management of Stroke. However, there are a small number of patients who show such stroke-like symptoms due to ascending aortic dissection (AAD) rather than intracranial artery occlusion. T-PA injection is contraindicated in cases of AAD. We recently experienced two such patients within a short period of time, and we report their cases herein.

## 2. Case  1

**Figure 1:**
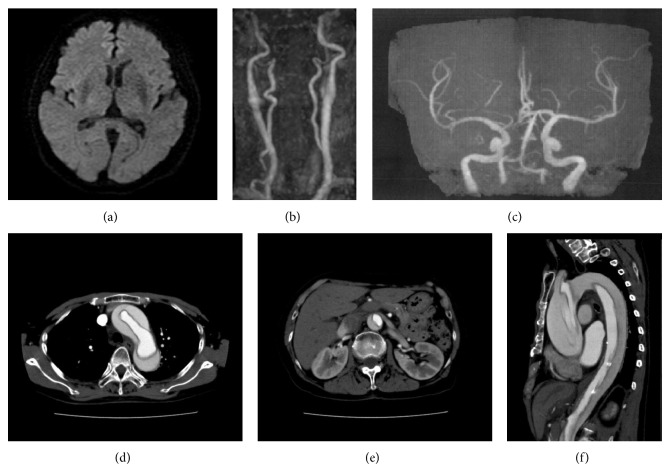
Axial head MRI-diffusion-weighted image (DWI) (a), carotid MR angiography (b), head MR angiography (c), axial chest to abdominal enhanced CT ((d), (e)), and sagittal view (f).

A 79-year-old woman with a history of hypertension, cerebral lacunar infarction, and goiter was transferred to our hospital presenting with loss of consciousness. Prior to presentation, she had come out of a swimming pool after training, experienced dizziness, and suddenly passed out. On arrival, her Glasgow Coma Scale (GCS) showed E1V1M4, and conjugate deviation to the right side and left monoparesis on the lower extremities were exhibited. Her NIHSS score was 23 points. She had an unexplainable low blood pressure (BP) of 85/45 mmHg and bradycardia of 41 beats per minute. Her emergency head computed tomography (CT) image was normal, her head magnetic resonance imaging-diffusion-weighted image (MRI-DWI) and head MR angiography (MRA) images did not show any fresh infarction (Figure 1(a)) or main branch occlusion, and neck MRA showed that both the internal and common carotid arteries were intact (Figures 1(b) and 1(c)). Her blood tests showed normal findings and no anemia. She was hospitalized in the intensive care unit (ICU) with a diagnosis of consciousness disturbance and hemiparesis of unknown etiology. Four hours later, her consciousness and hemiparesis improved; however, her BP remained low and we observed a decrease in urinary volume. She did not complain of chest or abdominal pain. We planned further examination to find the exact cause of her condition. A chest-to-abdominal enhanced CT was done and showed type A dissection from the ascending aorta to the bilateral common iliac artery (Figures 1(d), 1(e), and 1(f)). The patient was immediately transferred to a tertiary hospital to receive intensive care.

## 3. Case  2

A 72-year-old woman was found lying on her back grunting in the entrance of her house and was transferred to the ED at our hospital. On arrival, her neurological findings showed left hemiplegia and coma. Her BP was 110/76 mmHg measured in the right arm. While in the ED her respiratory condition gradually worsened in a manner suggestive of Cheyne-Stokes respiration, the emergency physician performed intratracheal intubation. Her NIHSS score was 23 points.

**Figure 2:**
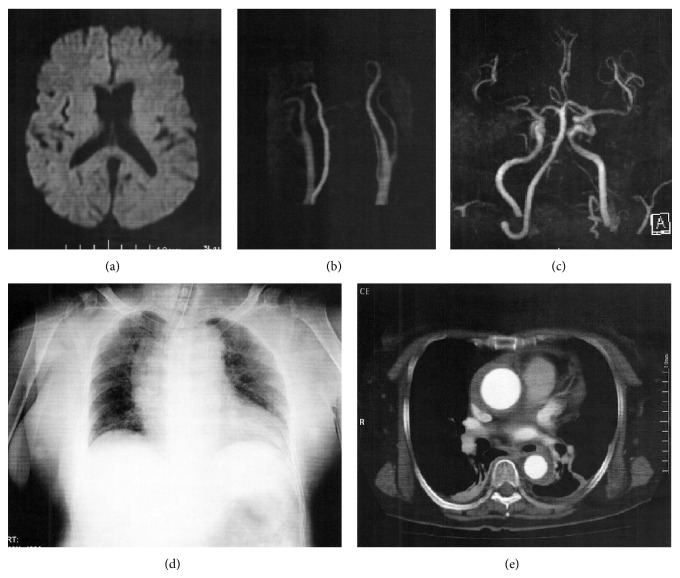
Axial head MRI-DWI (a), carotid MR angiography (b), head MR angiography (c), chest radiography (d), and axial chest to abdominal enhanced CT (e). Her pseudolumen had already been thrombosed and closed.

Emergency brain CT images showed normal findings and head MRI-DWI and head and carotid MRA images did not show signs of fresh infarction (Figure 2(a)) or main branch occlusion (Figures 2(b) and 2(c)). Although her chest radiography findings showed mediastinal widening, we did not take notice of it at that time (Figure 2(d)). Her blood tests showed normal findings. She was hospitalized in the ICU with consciousness disturbance and left hemiplegia of unknown etiology. On the following day, her consciousness disturbance was improved sufficiently that she could follow verbal orders. We decided to perform extubation. Following the extubation, her level of consciousness was almost clear, and she did not complain of chest symptoms, but she did experience dull left lumbar pain. To rule out the possibility of spinal infarction, we performed cervical spinal MRI-DWI, but the findings were normal. Her blood tests showed renal failure that became advanced in a single day. Finally, chest-to-abdominal CT was performed, and it showed AAD from the ascending aorta to the bilateral common iliac artery involving the left renal artery and renal infarction (Figure 2(e)). As the pseudolumen had already been thrombosed, she was referred to a cardiologist immediately. She received conservative management in the cardiovascular department. Her left hemiplegia was completely reversed after two weeks, and she left our hospital with no neurological deficits.

## 4. Discussion

Although MRI-DWI and MRA did not show acute stroke findings or main branch occlusion in either of these cases, in Case 1 we observed left monoparesis in one leg and conjugate deviation to the right side. We considered that two possible causes were involved in these symptoms. First, global cerebral ischemia may have caused hemiparesis and conjugate deviation as a neurological symptom on the side with a relatively weak blood supply, because the patient passed out and had continually shown marked low blood pressure. Nellamothu et al. reported that 13% of patients with type A dissection presented with syncope, and these patients showed a greater mortality rate because they were more likely to have cardiac tamponade and stroke [1]. Other studies have reported a frequency of 6–13% for type A dissection presenting with syncope [2, 3]. Further, in the second one of these studies, the patients with normal or low blood pressure made up more than 60% of all patients with type A dissection, while more than 70% of patients with type B dissection presented with a high BP of over 170 mmHg [3]. The second possible cause of the left monoparesis in Case 1 was ischemic neuropathy, since monoparesis occurred in only the left leg due to the extension of the dissection into the left common iliac artery, which could have interfered with the blood supply to the left leg. Unfortunately, we could not check whether symptoms of severe pain, coldness, numbness, or paresthesia were present in the lower extremity, as Gaul et al. reported, because of the patient's consciousness disturbance.

In Case 2, the diagnostic imaging findings showed left hemiplegia of unknown etiology. This finding did not agree with the clinical findings of spinal cord ischemia in the absence of symptoms such as anterior spinal artery syndrome or Brown-Sequard syndrome, and if such symptoms were present, irreversible neurological deficit might have occurred [4]. In this case, our speculation is that transient stenosis in the right common carotid artery (CCA), which progressed during aortic dissection, caused hemiparesis, and then the stenosis of the CCA improved gradually during the course, and, finally, the neurological symptoms disappeared. Two reports have described the observation of severe stenosis of CCA with thrombosed pseudolumen using carotid duplex ultrasonography (CUS), which was confirmed as CCA occlusion by MRA and was recanalized several days after diagnosis in both case reports [5, 6].

Both patients reported here came into our hospital presenting with clinical symptoms that were consistent with cerebrovascular diseases without any pain. The frequency of pain-free dissections has been shown to range from 5% to 15% [2, 4, 7]. In addition, in a study comparing a pain-free group with a group that experienced pain, it was shown that the frequencies of syncope, transient global amnesia (TGA), and other neurological symptoms were much higher in the group with pain [7]. Because such patients may not be able to complain of their pain due to consciousness disturbance or aphasia and memory loss, such examinations must be performed carefully [7]. Moreover, patients with pain-free dissection have worse outcomes than patients with painful dissection, at least diagnosis of the former condition requires so much time to be confirmed [7, 8]. Thus, it is important to remember that it may be difficult to diagnose patients with consciousness disturbance accurately because of the uncertainty of their clinical symptoms.

When a general practitioner or ER physician encounters patients with hyperacute stroke-like symptoms, it is important to first consider whether intravenous tPA injection can be used. Intravenous thrombolysis using t-PA (IV-tPA) therapy is contraindicated in cases with dissection [9]. The current Japanese guideline for IV-tPA mentions that neuroimaging should be performed using CT, but not necessarily MRI, before IV-tPA is performed. Therefore, patients with potential dissection may be included. In fact, 10 deaths due to IV-tPA in patients with dissection were reported in Japan before July 2007 [10]. In response to this finding, the Japan Stroke Society published an admonition of the doctors involved. In fact, many neurologists check MRI findings before administering IV-tPA, based on the belief that evaluation using only CT images is risky because of a lack of information.

Based on the results of the Safe Implementation of Treatments in Stroke-International Stroke Thrombolysis Registry (SITS-ISTR) [11] and the European Cooperative Acute Stroke Study III (ECAS III) [12], the time window of IV-tPA was extended from 3 hours to 4.5 hours after the onset of stroke symptoms in Japan in 2012, and in cases with indications for t-PA treatment the time window may be even greater. Therefore, we have to examine patients with hyperacute stroke symptoms carefully, keeping in mind the possibility that dissection may be present.

There are various examinations that can be easily performed in the ED in order to distinguish such cases from other patients with stroke symptoms. Chest radiography findings of mediastinal widening and abnormal aortic contour are considered to be specific to type A dissection, though they were not observed in one of our two cases. Based on the results of The International Registry of Acute Aortic Dissection (IRAD), such chest radiographic findings are absent in 37.4% of patients with type A dissection [3]. Even in the finding of absence of mediastinal widening or abnormal aortic contour, however, chest radiography may still be helpful.

As for type A dissection, the physical finding of a pulse deficit including a bilateral difference of pulse strength or loss of pulse is often helpful in obtaining a diagnosis. Bossone et al. reported that pulse deficits were noted in 30% of patients with type A dissection, and in-hospital complications and mortality were significantly higher in patients with pulse deficits [13].

The auscultation of cardiac murmur caused by aortic regurgitation (AR) was observed in 60–70% [14] of patients with type A dissection in IRAD, while it was documented in 44% of such patients in the study by Hagan et al. [3].

CUS examination is very helpful for the observation of CCA and ICA, and it usually demonstrates a subintimal dissection with a false lumen in type A dissection [5]. Furthermore, a thrombosed pseudolumen is observed as a long enhanced region like a hyperechoic plaque which does not cover the circumferential CCA cavity [5]. CUS is a noninvasive tool for diagnosis and a fast, easy-to-use examination that can be performed at bedside.

On the other hand, the D-dimer test is a useful and sensitive biomarker for the exclusion of dissection. Marill reported that the sensitivity of the D-dimer test was 94%, and the specificity ranged from 40% to 100% with a value of 0.5 *μ*g/mL defined as the threshold for positivity [15]. The specificity is not high because D-dimer also increases due to other thrombosis-related diseases, infection, and aging. Furthermore, the exclusion of dissection based on D-dimer at a threshold of 0.1 *μ*g/mL may be achieved with 100% sensitivity [16]. Accordingly, we would like to recommend the D-dimer examination to rule out potential dissection.

Currently, the exclusion of patients with stroke-like symptoms resulting from dissection is very difficult. In particular, patients with hyperacute stroke symptoms who had an episode of syncope or consciousness disturbance at the onset should be carefully examined before IV-tPA therapy.
